# Clinical complications and their management in a child with ataxia‐telangiectasia (A‐T): A case report study

**DOI:** 10.1002/ccr3.3581

**Published:** 2020-11-29

**Authors:** Marzieh Heidarzadeh Arani, Reza ArefNezhad, Javad Fathgharib, Asghar Aghamohammadi, Hossein Motedayyen

**Affiliations:** ^1^ Department of Pediatrics Faculty of Medicine Kashan University of Medical Sciences Kashan Iran; ^2^ Research and Development Department Exir Azma Salam Iranian Institute Tehran Iran; ^3^ Department of Anatomy School of Medicine Shiraz University of Medical Sciences Shiraz Iran; ^4^ Research Center for Immuynodeficiency Tehran University of Medical Sciences Tehran Iran; ^5^ Autoimmune Diseases Research Center Kashan University of Medical Sciences Kashan Iran

**Keywords:** ataxia‐telangiectasia, recurrent pneumonia, The *ataxia‐telangiectasia mutated (ATM)* gene

## Abstract

Ataxia‐telangiectasia (A‐T) is known as an uncommon autosomal recessive disorder associated with recurrent infections and other clinical complications. The management of its complications can improve life quality of patients.

## Graphical abstract

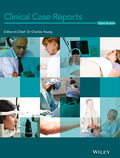

## 1 INTRODUCTION

Ataxia‐telangiectasia (A‐T) is known as an uncommon autosomal recessive disorder related to the nervous system which involves several systems. It is described by telangiectasia, immunodeficiency, ataxia, radiosensitivity, and susceptibility to various infections and hematologic malignancies.^1^ This disorder is largely related to mutations in the *ataxia‐telangiectasia mutated (ATM)* gene.^2^, ^3^ A‐T affects 1 or 2 per 100 000 subjects globally, and its prevalence in men is similar to women.^4^, ^5^ The morbidity of A‐T may contribute to pulmonary disease, immunodeficiency, and dysphagia.^2^ Moreover, various infections are responsible for its morbidity and mortality.^6^ The *ATM* gene codes for a ATM serine/threonine kinase, which is recruited and activated by DNA double‐strand breaks, and plays a pivotal role in the reconstruction of fractured DNA during the differentiation and development of the cells such as B and T cells.^7^, ^8^ In addition to the role of *ATM* mutations in susceptibility to A‐T, genetic variations in this gene contribute to the development of different cancers such as lymphoma and breast cancer.^7^, ^9^ Some relationships have been reported between A‐T and other disorders such as hyperimmunoglobulin (HIgM) syndrome and dystonia,^10^ but the associations of A‐T with other disorders are not well identified yet.

Regarding the fact that there is not notable advance in A‐T treatment, early diagnosis of A‐T may have a fundamental role in controlling disease complications and thereby improving life quality of patients.^5^ In this report, we reported a pediatric patient with A‐T to better diagnosis and management of A‐T complications.

## 2 CASE HISTORY

Our case is an 8‐year‐old boy who had recurrent respiratory infections and other health problems. He was born via elective cesarean section from a mother who delivered at 41‐week gestation. His birth weight was 3250 g. His parents had consanguinity. He had family history of A‐T in his mother's uncle's children. His umbilical cord dropped at 11 days of age. The routine vaccination was done, and he was nourished with mother's milk. Thus, growth and weight gaining were normal. Our case had mouth aphthous during the first year of age and was hospitalized in Kashan Shahid Beheshti hospital owing to pneumonia for one week. He suffered from ataxia during sitting at 15 months of age. Magnetic resonance imaging (MRI) was carried out to determine cause(s) of ataxia. The result of MRI was normal. After two months, he had ataxia during walking. The patient was hospitalized due to severe fever and neutropenia at 18 months of age and discharged from Kashan Shahid Beheshti hospital after a recovery period. During these years, our case was also admitted to Kashan Shahid Beheshti hospital three times because of fever and suppurative rhinitis. When the patient was three years old, he suffered from petechial hemorrhage, thrombocytopenia, and mouth bleeding. Idiopathic thrombocytopenic purpura (ITP) was considered, and he was treated with intravenous immunoglobulin (IVIG). However, patient had petechial hemorrhage all over the body after ten days. Bone marrow (BM) aspiration was done to detect the cause(s) of thrombocytopenia. The result was normal. Having considered that thrombocytopenia may associate with splenomegaly, computerized tomography (CT) scan imaging was performed. Spleen size was normal. Thrombocytopenia started to recover gradually following the treatment with IVIG and corticosteroids. At 3.5 years of age, our case experienced acute otitis media, which was treated with antibiotic therapy (meropenem and vancomycin) and ventilation tube (VT). During these years, he also had several episodes of various infections (pneumonia and otitis) and acute gastroenteritis, which were treated with co‐amoxiclav, azithromycin, and tavanex. The patient suffered from herpetic gingivostomatitis, parotiditis, and chickenpox at age four years. Moreover, he had otitis and neutropenia again. Therefore, he was hospitalized in Kashan Shahid Beheshti hospital for one week. Some laboratory features of patient were investigated (Table 1). Based on laboratory data, HIgM syndrome was initially diagnosed. Antimicrobial and antiviral therapies and IVIG administration were used to treat infectious complications and then discharged. When our case was six years ago, he had nystagmus accompanied by severe telangiectasia and ataxia. He was treated with behavior therapy. These clinical manifestations along with family history of A‐T suggested that our case should be genetically studied to determine a possible defect in the *ATM* gene. The genetic analysis revealed that our case had A‐T (Table 2). After certain diagnosis of A‐T, antibiotic prophylaxes, IVIG administration, and specific food regimes were considered as a suitable approach to control clinical status of patient. Our case is currently alive and in acceptable health condition.

**Table 1:** Laboratory characteristics of a patient with A‐T

Biochemistry	Cell numbers or values	Total counted cells or normal ranges
ALT	75	Up to 41
AST	78	Up to 37
ALP	573	98‐279
GGT	26	100‐500
AFP	275.7	0.1‐10
Immunology		
Anti‐tetanus Ab	<0.1	<0.1
Anti‐diphtheria Ab	<0.1	<0.1
Anti‐HCV Ab	Negative	
Anti‐HAV Ab	Negative	
Anti‐HBS Ab	94.2	10‐100 weakly positive
IgM	724	37‐224 mg/dL
IgA	2	25‐154 mg/dL
IgE	0	1.53‐11.4 mg/dL
IgG	27	386‐1470 mg/dL
CD3+ cell population	69%	67%‐69%
CD4+ cell population	40%	39%‐40%
CD8+ cell population	19%	25%‐27%
CD19+ cell population	10%	14%‐15%
CD56+ cell population	10%	10%‐15%
CD16+ cell population	10%	10%‐15%
Stool examination		
RBC	0‐1	1‐2
WBC	4‐6	1‐2
Fat drop	Not seen	
Cryptosporidium	Positive	

**Table 2:** Genetic analysis of the *ATM* gene

Gene/transcript (RefSeq)	Variant location	Variant	Chromosome position	Zygosity	Related phenotypes	OMIM number	Inheritance pattern	Variant classification	Reference
ATM NM_000051.3	Exon 26	c.3895delG p.A1299Pfs*50	Chr11:108,155,101	Hom	Ataxia‐telangiectasia	208900	AR	Pathogenic	1
					Susceptibility to breast cancer	114480	SMu, AD		
					Lymphoma, B cell non‐Hodgkin, somatic	‐	‐		
					Lymphoma, mantle cell, somatic	‐	‐		
					T‐cell prolymphocytic leukemia, somatic	‐	‐		

## 3 DISCUSSION

Ataxia‐telangiectasia (A‐T) is a genetic neurodegenerative disorder described by several problems such as ataxia, telangiectasia, failure in motor ability, unusual eye movement, immunodeficiency, pulmonary disorder, and susceptibility to recurrent infections due to the mutation in the *ATM* gene on the chromosome 11q22.23.^1^, ^2^, ^11^ These clinical complications play indispensable role in the morbidity and mortality of the disease.^6^ In the present study, we investigated a child with A‐T who had recurrent hospital admission due to repetitive infections, hematologic disorders, and neurological problems.

Previous studies have indicated that some clinical complications are mediated by immune disorders in A‐T patients.^12^ It is shown that inflammation participates in the pathogenesis of the disease. inflammatory cytokines had the increased levels in A‐T patients.^13^ These increased levels can be involved in inflammatory diseases such as suppurative rhinitis, gastroenteritis, acute otitis media, and parotiditis, which were observed in our case. In line with the role of immunodeficiency, some reports have suggested that hematologic disorders such as thrombocytopenia may correlate to immune disorders in A‐T patients.^12^, ^14^ Moreover, our patient had hematologic problems which were treated with IVIG and corticosteroids. Our observation provides further confirmation which immune disorders have an important role in the pathogenesis of some clinical complications of A‐T.

One of the characterizations of A‐T is cerebellar neurodegeneration.^15^ In addition to impact of neurological disorders in movement problems, it is revealed that these disorders may participate in development of pulmonary complications in A‐T.^2^ The clinical findings of our patient were consistent with previous reports pointing to neurological involvement in A‐T.^15^ Nervous system disorders in our case included ataxia and nystagmus. Several studies have mentioned to the differences of the neurogenesis kinetics between A‐T patients and healthy subjects, due perhaps to the *ATM* mutation.^15^

The reduced productions and impaired functions of IgA, IgG are considered as the identified characteristics of A‐T.^16^ Our case also showed the reduced values of IgG, IgA, and IgE accompanied by the decreased numbers of lymphocytes (CD8^+^ cells and CD19^+^ cells) in comparison with healthy subjects,^17^, ^18^ which are other clinical features observed in A‐T patients.^12^, ^16^ Moreover, A‐T patients may have HIgM syndrome and various viral and opportunistic infections.^2^ In agreement with findings, our patient suffered from recurrent pneumonia, chickenpox, and HIgM syndrome.

## 4 CONCLUSION

Our findings indicated that patients suffering from recurrent infectious diseases and other health problems should be investigated to determine possible defect(s) in the *ATM* gene. In addition, our case suggests that a patient with A‐T may have clinical and laboratory features of HIgM syndrome appeared before other A‐T complications. Therefore, early diagnosis of A‐T has a fundamental role in managing clinical complications of A‐T and thereby improving life quality of patient.

## CONFLICT OF INTEREST

None declared.

## AUTHOR CONTRIBUTION

Marzieh Heidarzadeh carried out some of the experiments. Reza ArefNezhad participated in the design of the experiments. Javad Fathgharib collected the laboratory findings and performed some experiments. Asghar Aghamohammadi participated in the design of the experiments. Hossein Motedayyen drafted the manuscript and participated in the study design. All authors read and approved the final manuscript.

## ETHICAL APPROVAL

This study was approved by the Ethics Committee of Kashan University of Medical Science.

## Data Availability

All data generated or analyzed during this study are included in this published case report.
